# Using Kernel Method to Include Firm Correlation for Stock Price Prediction

**DOI:** 10.1155/2022/4964394

**Published:** 2022-04-05

**Authors:** Hang Xu

**Affiliations:** Shanghai University of Finance and Economics, Shanghai, China

## Abstract

In this work, we propose AGKN (attention-based graph learning kernel network), a novel framework to incorporate information of correlated firms of a target stock for its price prediction in an end-to-end way. We first construct a stock-axis attention module to extract dynamic and asymmetric spatial correlations through the kernel method and a graph learning module into which more accurate information can be integrated. An ensemble time-axis attention module is then applied to learn temporal correlations within each stock and market index. Finally, we utilize a transformer encoder to jointly attend to obtain information from different levels for correlations' aggregation and prediction. Experiments with data collected from the Chinese stock market show that AGKN outperforms state-of-the-art baseline methods, making up to 4.3% lower error than the best competitors. The ablation study shows that AGKN pays more attention to hidden correlation between stocks, which improves model's performance greatly.

## 1. Introduction

Stock prediction has drawn a lot of attention from researchers for a long time. Accurate stock prediction can help investors make appropriate decisions, acting as a meaningful index of portfolio allocation [1]. Efficient market hypothesis (EMH) assumes that all the participants in the market are informationally efficient and that all deals are traded at fair values [2], which mean that the stock market can respond to new information rapidly and stock prices are full representations of market. Therefore, it is impossible to predict stock prices under EMH's framework. However, the rationality of this framework is questionable in reality. Behavioral economists then propose adaptive market hypothesis (AMH) to revise EMH [3]. AMH takes interaction between market and participants into consideration and indicates that it is feasible to obtain excess return through information asymmetry, which implies that the stock is predictable.

Traditional time-series forecasting methods include the AR model, the ARMA model, and the ARIMA model which take single time series as input and make prediction through fitting. Those models can only extract the linear relationship of time series, but the correlation in financial data is always nonlinear [4]. Recently, machine learning and deep learning have become gradually popular in stock prediction. Lee et al. use SVM to extract features and make prediction [5]. Ballings et al. forecast stock price based on ensembles' algorithm including random forest, Adaboost, and kernel factory [6]. Such methods for individual prediction focus on temporal prices pattern of each stock, ignoring the interaction between stocks.

Some previous works utilize models based on LSTM and Markov random fields (MRF) for correlated stocks prediction [7, 8]. Qin et al. propose dual-stage attention-based recurrent neural network (DA-RNN) to learn relationships between stocks automatically [9]. Yoo et al. propose data-axis transformer with the multilevel context (DTML) model for stock movement prediction [8]. DTML utilizes attention-based LSTM and multihead attention for time-axis and data-axis relation extraction, respectively. However, the data-axis module follows time axis, which means that time-axis attention will influence model performance greatly. This will lead to unstable results and poor generalization ability of the model. One of the key challenges for multiple stocks prediction is “how to correlate historical price data both in stock axis and time axis”. Many researchers choose to use graphing neural networks (GNNs) to address this issue because GNN can effectively extract correlation from nodes [7, 10, 11]. However, GNN has the following limitations. First, it requires graph structure as prior knowledge, which is always not defined in financial data. Second, those predefined structure between stocks is fixed, while true correlations keep changing with market. In order to address these issues, the kernel method-based model has been applied. The kernel method plays an important role for data presentation prior to neural network [12]. After mapping features such as vectors and matrix to high-dimensional space by the kernel method, the well-honed linear model can also achieve high performance. This method has a great advantage: it is nonparametric, which is crucial for computation complex. And it can enhance the expressiveness of raw data and extract useful information from mass data. And using correlated stocks for prediction may introduce too many parameters, as well as information noise. We take advantage of the kernel method to solve those problems.

**Figure 1:**
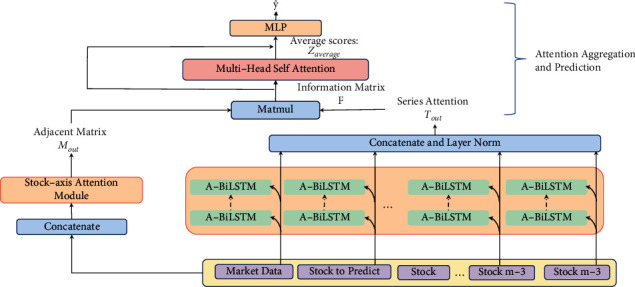
The overall architecture of AGKN, which consists of three main modules. First, stock-axis attention module computes the adjacent matrix *M*_out_. Second, several A-BilSTMs extract temporal attention within each stocks and concatenate them as *T*_out_. Finally, transformer encoder is used to aggregate them and make prediction.

In this study, we propose an end-to-end framework, AGKN (attention-based graph learning kernel network), for correlated stocks prediction. As demonstrated in Figure 1, our model consists of three parts: stock-axis attention generation module, time-axis attention generation module, and attention aggregation module. In stock axis, correlated stocks' data are projected into an reproducing kernel Hilbert space (RKHS) with the learnable kernel method. A novel adjacent matrix extraction layer is further proposed for stock-axis attention capture based on transformed data. In time-axis, attention-based bidirectional LSTM (A-BiLSTM) is applied to obtain the attention scores. Ultimately, an aggregation module is used for attention scores' aggregation and final prediction. In AGKN, asymmetric and dynamic attention scores are generated by the adjacent matrix extraction layer, avoiding the given disadvantages of GNN, and market data are included to respond to changes in macroenvironment flexibly. The kernel method acts as a data intensifier and significantly improves model performance.

Our contributions are summarized as follows:

- To our best knowledge, this is the first attempt to study correlated stock prediction based on the kernel method. Because too many features as well as noises exist for correlated stocks' prediction, this nonparametric method can enhance raw data and reduce model parameters to prevent overfitting.
- We proposed a novel framework, AGKN, for correlated stocks' prediction in an end-to-end way, which can obtain dynamic data relationships both in stock axis and time axis.
- We conducted extensive experiments to evaluate AGKN. Experimental results show that AGKN outperforms the state-of-the-art methods. Furthermore, we analyze the stock-axis attention scores, and it proves that our novel architecture is effective in correlation extraction.

The reminders of this paper are organized as follows. In Section 2, we summarize relevant works in correlated stock prediction and kernel method. In Section 3, we present the main methodologies and components of AGKN. In Section 4, we show the experiment settings, results, and analysis. The conclusion will be given in Section 5.

## 2. Related Works

### 2.1. Graph Neural Network and Attention Mechanism for Correlated Stock Prediction

Most stocks can be clustered according to industries [13]. Stocks in the same industry have similar price trends in the long term except short-term random disturbance, which acts as a reliable hint for investors. However it remains a challenge to obtain correlation from historical data. Chen et al. take common shareholders in different stocks as nodes and use graph convolutional network (GCN) to capture correlation, which cannot fully represent real-time market changes due to lags of shareholder information update [11]. Cao et al. combine graph Fourier transformer and discrete Fourier transformer in spectral temporal graph neural network (stem-GNN) and use stem-GNN to model interseries and intraseries correlation [10]. Li et al. encode lead-lag relationships between stocks using weighted higher-order Markov random fields (MRFs) and treat them as nodes of MRFs [7]. However, they all require graph structure as prior knowledge, which is not defined in financial data. In fact, spatial-temporal graph neural networks are probably the most suitable type of GNN for multivariate time series, which takes multivariate time series and an external graph as input [14]. In addition, using graph attention network (GAT) for node-level and semantic-level relation capture has achieved good results [15, 16].

Wu et al. propose graph learning module to automatically extract the unidirected relations among multivariate data. A novel mix-hop propagation layer and dilated inception layer are further applied to capture the spatial and temporal dependencies within each time series [17]. However, because financial data are always high noise, frequent alternations of those modules in the proposed framework may exacerbate noise propagation to reduce the model accuracy.

In order to make full use of correlated stocks data, we propose a novel framework to obtain both temporal and spatial attention from raw data in a synchronized rather than step-by-step way. Because financial data are marked by leptokurtic [18], volatility clustering [19], and low signal-noise ratio (SNR) [20], we need to design dedicated modules for specific tasks based on raw data.

### 2.2. Kernel Method in Neural Networks

The kernel method is closely related to infinitely wide neural networks. Neal and R.M. point out that a single hidden layer neural network with independent, identically distributed random parameters, in the limit of infinite width, is a function drawn from a Gaussian process (GP) [21]. Recent works extend it to deep neural networks and convolutional neural network (CNN) [22, 23]. Jacot et al. believe that least squares regression based on neural tangent kernel (NTK) equals to infinitely wide neural networks [24]. Lee et al. put forward that optimizing the last layer of an infinite wide neural network is equivalent to a Gaussian process based on neural network [25]. Therefore, the kernel method can be applied as one way to improve the performance of deep neural networks. Mairal et al. propose convolutional kernel network (CKN) to learn to approximate the kernel feature map on training data and present the backpropagation method in CKN, bridging a gap between the kernel method and the neural network [26, 27]. Chen et al. generalize convolutional kernel networks to graph-structured data by representing graphs as a sequence of kernel feature maps. However, none of them focus on financial data, which have distinctive characters.

Liu et al. propose the recurrent convolutional neural kernel (RCNK) model, which learned complementary features from historical data and text data, to predict the stock price movement [28]. RCNK constructs an explicit kernel mapping layer to replace fully connected layers to reduce the number of parameters and maintain accuracy. However, RCKN fails to take correlation between stocks into consideration, which is an important character in financial domain.

Generally, a positive defined kernel implicitly induces reproducing kernel Hilbert space (RKHS) and transforms the linear model in RCKS to nonlinear model in input space. This method can reduce the number of parameters in architecture and enhance data presentation. Therefore, merging kernel method and deep neural network can decrease the computation complexity and the risk of overfitting. Besides, through transformation of the learnable kernel method in network, information hidden in raw data is apt to be extracted in the following modules to improve model accuracy.

## 3. Proposed Approach

### 3.1. Overview

**Figure 2:**
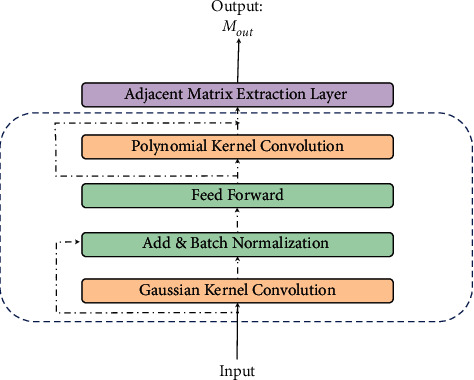
Ensemble stock-axis attention module. The Gaussian and polynomial kervolution are used to transform data.

AGKN is based on the kernel method and GNN, in which correlated stocks' information is used coherently to improve model performance. As illustrated in Figure 1, AGKN consists of stock-axis attention module, time-axis attention module, and aggregation module. To discover hidden correlation among stocks, our stock-axis attention module utilizes a novel layer to extract adjacency matrix. Prior to it, we use the learnable kernel method, instead of a fixed one, to transform data, acting as a data intensifier. As a result, the data intensifier can adapt itself to uncertain market circumstances. The learnable way makes it easy to extract correlation, which addresses the limitations of previous works concluded in Section 2.1.  Figure 2 presents the framework of stock-axis attention module. In time axis, attention-based LSTM is used to capture relation within historical data of each stock. Instead of proceeding in lockstep, the operations in both directions are synchronized. It is because financial data are always low-SNR (signal-to-noise ratio) and noise tends to accumulate and propagate in feedforward. The synchronized structure can extract more useful context from raw data and reduce noise generation at the same time, which avoids drawbacks we put forward in Section 2.2. Finally, two attention maps are fed into output module, in which we apply transformer encoder [12, 20] for attention aggregation and a fully connected layer for prediction. In this way, temporal and spatial correlations are effectively combined and AGKN performance advances greatly. In more detail, the core components of our model are illustrated in the following.

### 3.2. Convolutional Kernel Layer

We first elaborate on the general theory of kernel convolution (kervolution) developed in [26]. The kervolution learns to map the given data into a reproducing kernel Hilbert space (RKHS) with trainable parameters in neural network. In RKHS, we can find a subspace to divide transformed data. Given a set *χ*, a positive definite kernel K: *χ*×*χ*⟶ℝ implicitly defines an RKHS H and a mapping *φ*: *χ*⟶ℋ:

(1) $$\begin{array}{c} K_{1}\left(x,x^{\prime }\right)={\langle \varphi \left(x\right),\varphi \left(x^{\prime }\right)\rangle }_{Η}, \end{array}$$

where 〈., .〉 denote the convolutional operator, *x*, *x*′ ∈ *χ*. Specifically, consider two feature matrix *x*, *x*′ ⊂ *ℝ*^*p∗q*^, where *p* and *q* represent width and height. The kernel method *K*_1_ is defined as

(2) $$\begin{array}{c} K_{1}\left(x,x^{\prime }\right)=\left\|x\right\|\left\|x^{\prime }\right\|\kappa_{1}\left(\langle \frac{x}{\left\|x\right\|},\frac{x^{\prime }}{\left\|x^{\prime }\right\|}\rangle \right), \end{array}$$

where ‖·‖ denote the usual Euclidean norm and *κ*_1_ is a dot product kernel on the sphere, and we require *κ*_1_ to be smooth enough and its Taylor expansion have nonnegative coefficients to ensure positive definiteness [12]. Specially, the kernel method can be recursively used to produce multilevel features by turning various pixel attributes into patch level, which is useful for nonlinear representation of neighborhoods [29].

Given a convolution filter *ω* ∈ *R*^*k*·*k*^ and a feature map Ω ⊂ *ℝ*^*p∗q*^, x ⊂ Ω, a kervolution is defined as

(3) $$\begin{array}{c} K_{1}\left(x,\omega \right)=\left\|x\right\|\kappa_{1}\left(\langle \frac{x}{\left\|x\right\|},\omega \rangle \right). \end{array}$$

**Figure 3:**
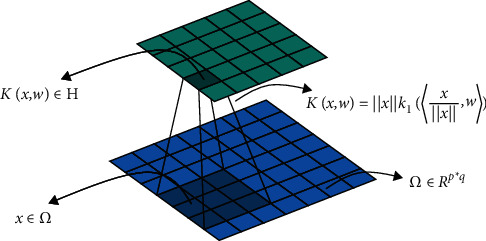
Kervolution for one channel.

Figure 3 illustrates the concrete operation for a single channel.

Polynomial kervolution is defined as

(4) $$\begin{array}{c} K_{p}\left(x,w\right)={\left(x^{T}w+c_{p}\right)}^{d_{p}}=\sum_{j=0}^{d_{p}}c_{p}^{d_{p}-j}{\left(x^{T}w\right)}^{j}, \end{array}$$

where *w* is the convolution filter, *x* is sliding windows for *w*, and *d*_*p*_ ∈ *Z*^+^and*c*_*p*_ ∈ *R*^+^ are hyperparameters. Dai et al. show that polynomial kernel performs well for natural language processing (NLP) when *d*_*p*_=2. Wang et al. find *d*_*p*_=3 is more appropriate for matrix recognition [30, 31].

Gaussian kervolution (RBF) is an extension to infinite dimensions:

(5) $$\begin{array}{c} K_{G}\left(x,w\right)=\exp\left(-\frac{\alpha }{2}{\left\|x-w\right\|}_{2}^{2}\right), \end{array}$$

where ‖·‖_2_ denote unit ℓ-2 norm and *α* is a hyperparameter. From Taylor expansion, RBF extends the kernel method to infinite dimensions:

(6) $$\begin{array}{c} K_{G}\left(x,w\right)=\exp\left(-\frac{\alpha }{2}\left({\left\|x\right\|}_{2}^{2}+{\left\|w\right\|}_{2}^{2}\right)\right)\sum_{\text{i}=0}^{\infty }\frac{{\left(x^{T}w\right)}^{i}}{i!}. \end{array}$$

Additionally, complexity of kervolution is normally O(n) as the same as the inner product of convolution [26]. Therefore, by applying kervolution, AGKN can maintain low computational complexity while focusing on different levels as much as possible.

### 3.3. Stock-Axis Attention Generation

Some graph-based prediction models follow three main steps [10]. First, historical stock prices are fed into the sequence layer to output node embedding for each stock. These outputs are used to capture short-term price trend. Then, an adjacency matrix is created based on some other open graph sources such as Wiki-data or semantic text data on stock forum. Finally, a graph neural network is employed to combine the node embeddings and adjacency matrix as well as to make prediction. Such predefined and multidata-based models are likely to confuse information hidden in stock market and fail to capture dynamic correlation between stocks in different time steps [32] Those drawbacks restrict effective spatial dependencies learning of models. In this phase, we propose a novel matrix extraction layer to extract dynamic spatial dependencies among nodes (stocks) based on transformed data with the kernel method. Furthermore, influence between stocks is usually asymmetric. For instance, small firms' stock prices tend to be more responsive to changes in other stocks prices than large firm does [33]. Thus, the influence of large firm's stock price on small firm is relatively greater than reverse influence. Our data-driven method addresses these problems, extracting dynamic and asymmetric adjacent matrix automatically, which is illustrated as follows:

(7) $$\begin{array}{c} M_{1}=\tanh\left(EX\right), \end{array}$$

(8) $$\begin{array}{c} M_{2}=\text{SoftMax}\left(\tanh\left(M_{1}BM_{1}^{T}\right)\right), \end{array}$$

(9) $$\begin{array}{c} M_{\text{out}}=\alpha M_{2}+\left(1-\alpha \right)P, \end{array}$$

where *X* denotes feature matrix transformed by kernel method, *E*, *B*, and *α* are learnable parameters, and P represents Pearson correlation coefficient matrix.

Equation (7) is expanded as

(10) $$\begin{array}{c} M_{1}\left[i,j\right]=\tanh\left(\sum_{j=0}^{m}e_{i,j}x_{j,i}\right). \end{array}$$

From this perspective, items in matrix *M*_1_ can be viewed as weight-average impact of other stocks and market on specific stock in specific time step. In this way, interactions among nodes are taken into consideration in each time step, which enhances the expressiveness of the model. The asymmetric property of our proposed method is achieved by equation (8), which summarizes information of all interacting nodes through hidden edges in entire rolling windows. B acts as a weight matrix in this equation and SoftMax activation function is employed as regularize of the adjacent matrix.

### 3.4. Time-Axis Attention Generation

Another important part for price prediction is how to obtain temporal dependencies within historical data. To answer this question, we apply LSTM-based model. LSTM addresses the vanishing gradient problem and has stronger memory within time series. Given feature vectors and state vectors, a LSTM cell computes the current state vectors and feeds them into the next cell. Zhou et al. combined attention mechanism and LSTM to capture different information in a sequence, which can handle time series well so that we introduce it into AGKN [34]. The structure of this model is simple and easy to understand, which is shown below.

**Attention LSTM:** let H be a matrix consisting of the hidden states in each time-step [*h*_1_, *h*_2_, ..., *h*_*T*_], where *T* represents the sequence length, and the time-axis attention is computed as

(11) $$\begin{array}{c} M=\tanh\left(H\right),\\ \alpha =\text{SoftMax}\left(w^{T}M\right),\\ T_{\text{out}}=H\alpha^{T}, \end{array}$$

where *w* is a learnable parameter. The detailed implementation is shown in [34].

### 3.5. Multilevel Attention Aggregation

Finally, AGKN integrates stock-axis attention and time-axis attention for prediction. In this module, we employ multihead attention to tackle this task for its ability to jointly attend to information from different levels. Given attention matrixes in two directions, *T*_out_ and *M*_out_, the input of attention *F* is defined as

(12) $$\begin{array}{c} F=M_{\text{out}}T_{\text{out}}. \end{array}$$

Thus, multihead attention learns to extract correlation between stocks from hybrid attention matrix *F* and make final prediction.

Transformer encoder used for final prediction: the process of self-attention is shown as follows:

(13) $$\begin{array}{c} Q=FW_{Q},\\ K=FW_{K},\\ V=FW_{V},\\ Z_{0}=\text{SoftMax}\left(\frac{QK^{T}}{\sqrt{d_{k}}}\right)V, \end{array}$$

where *d*_*k*_ denotes sequence length of time series and *W*_*Q*_, *W*_*K*_, and*W*_*V*_ are learnable parameters.

In order to capture various information, multihead attention defines N sets of *W*_*Q*_, *W*_*K*_, and*W*_*V*_ to obtain N attention scores *Z*_1_, *Z*_2_, ..., *Z*_*N*_. These scores focus on different aspects of data. The attention output is the average of all attention heads. Then, a residual connection is applied to avoid vanishing gradient:

(14) $$\begin{array}{c} Z=Z_{\text{average}}+F, \end{array}$$

where *Z* gives us some hints of correlation between stocks in an asymmetric way. Finally, several fully connected layers and activation functions are used for final prediction.

## 4. Experiments, Results, and Discussion

In this section, we present our experiment setting and results and give detailed analysis. We compare the performance of AGKN with other baseline models. Furthermore, to verify the importance of the kernel method and the adjacent matrix extraction layer in AGKN, we remove the corresponding modules and find that corresponding models perform poorly from different perspectives. Finally, we analyze the results from stock-axis attention generation to emphasize its function again.

### 4.1. Dataset

We collect daily data of 13 stocks and 1 market index from Shanghai and Shenzhen stock markets in China from 3 January 2017 to 22 October 2021. The transaction data of each trading day are taken as raw data, including opening price, closing price, the highest price, the lowest price, trading volume, and amount.  Table 1 summarizes statistics of dataset. The data are from https://tushare.pro/(access on 1 November 2021).

We move a lag window with size of *h* time steps to construct the feature matrix and label it with the next day's closing price. We segment data with the first 70% of the date for the training dataset and final 30% for test data.

### 4.2. Comparative Models

Firstly, to evaluate the effectiveness of stock and time attention module, we take AGKN without them separately as trial objects. Furthermore, we remove the kernel method and adjacent matrix extraction layer, respectively, and include them in the trial group. In addition, kervolutional neural network (KNN) [31], A-BiLSTM [34], and long- and short-term time-series network (LSTNet) [35] will be used as baseline models for comparative experiments.  Table 2 shows the abbreviations of comparative models.

### 4.3. Metrics and Hyperparameters

We evaluate the result of stock price prediction by two metrics: mean squared error (MSE) and root mean square percentage error (RMSPE). These two metrics are always used to measure the quality of regression problems, which is defined as follows:

(15) $$\begin{array}{c} \text{MSE}=\frac{1}{N}\sum_{i=1}^{N}{\left(y_{i}^{\text{pre}}-y_{i}^{\text{true}}\right)}^{2},\\ \text{RMSPE}=\sqrt{\frac{1}{N}{\sum_{i=1}^{N}\left(\frac{y_{i}^{\text{pre}}-y_{i}^{\text{true}}}{y_{i}^{\text{pre}}}\right)}^{2}}, \end{array}$$

where *y*^pre^ is predicted price and *y*^true^ is true price.

After attempting many times, AGKN's hyperparameters are shown as follows. Lag window size *h* is 20, Gaussian kervolution's kernel size is 4, polynomial kervolution's *d*_*p*_ is 4, the number of epochs is 500, the number of A-BiLSTM's layers is 2, and optimizer is Adam optimizer with both learning rate and regularization weight being 0.01.

### 4.4. Results

**Figure 4:**
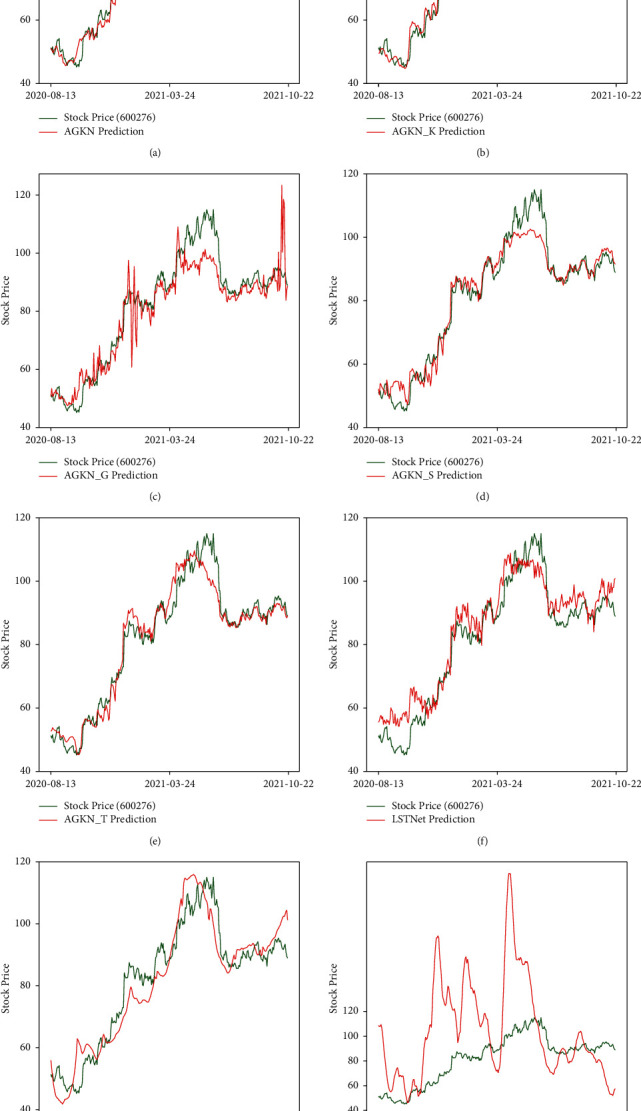
Comparison of experiment results. (a) AGKN. (b) AGKN-K. (c) AGKN-G. (d) AGKN-S. (e) AGKN-T. (f) LSTNet. (g) A-BiLSTM. (h) KNN.

We test AGKN and its competitors. The performance is evaluated by MSE and RMSPE.  Table 3 shows the performance of the different models, and Figure 4 visualizes the predicted price.

### 4.5. Ablation Study

From the results, we can conclude that

**Figure 5:**
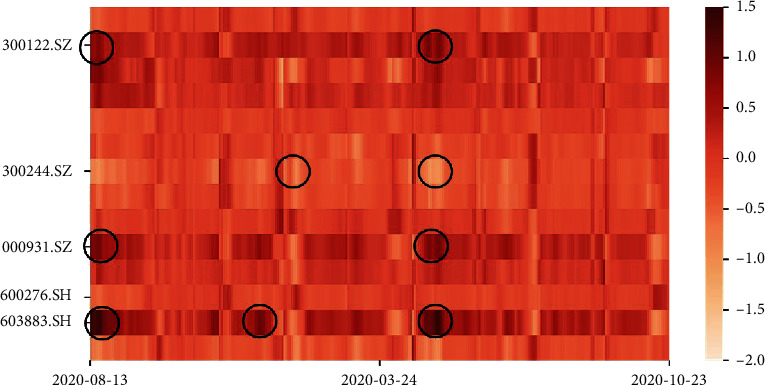
Concatenated stock-axis attention map for single stock: 600276.SH.

1. Each module improves the model performance, and AGKN with complete structure produces the best results. AGKN outperforms AGKN-G and AGKN-S greatly, which means that *G* and S are more important in the whole framework. As for AGKN-K, AGKN do not show a huge improvement from it. Actually, as we can see from Figure 4, AGKN and its derivatives all perform bad in the area within red box of AGKN's and AGKN-K's result figures. However, in this area, there is an obvious synergetic effect between predicted and realistic trend for the AGKN model. It means that the kernel method we utilized can make the model pay more attention to hidden information which cannot be extracted with other model. That is what we expected the kernel method to do! AGKN-T's performance does not differ greatly from AGKN. However, its result shows distinctive time lag. In other words, the predicted price is close to its prices of previous few days, which is not what we want. Actually, the time lag is as it should be because *T* module is used to extract time correlation. Without this module, time-axis information cannot be used effectively, causing the time lag.
2. AGKN outperforms A-BiLSTM and LSTNet, which means it is far from enough to focus only on temporal correlation within historical data. We need to pay more attention to spatial dependencies. On the contrary, KNN's performance is poor. It is because KNN is of numerical instability. If we apply it alone, we will always fail on training.
3. Figure 5 shows the concatenated attention scores between stocks in Table 1 with respect to target stock (600276.SH). The scores represent how much target stock considers others at each moment. From the figure, we can observe three main points. First, certain stock scores change smoothly over time. This is natural because stock market is continuous so that the property of a stock does not change instantly at a moment. Second, attention scores vary greatly among stocks both at single time step and in the whole time period. 300122.SZ, 300244.SZ, 000931.SZ, and 603883.SH are especially crucial for target stock. The variety stresses the significance of correlated stocks prediction. Third, attention scores for one stock differ greatly in different periods with large gap. This is because stocks are always volatility clustering and their correlations always change greatly along with time, which corresponds to dynamic character we emphasized above.

## 5. Conclusions

In this study, we proposed a hybrid model named attention-based graph learning kernel network (AGKN) for correlated stock prediction. AGKN effectively extracts attention both in time axis and data axis without any prior knowledge. By mapping raw data into RCHKs, we proposed a novel way to exploit the correlations among multiple stock data. The experiment results prove the effectiveness of each section in AGKN. Furthermore, our model outperforms a variety of stock prediction methods and acts as a development of the multivariate stock movement prediction in [8].

## Figures and Tables

**Table 1:** Description of data. The mean and variance of closing, highest, and lowest prices are similar to open, so they are not displayed here. We aim to predict the closing price of 600276.SH. Market index is 930767.CSI.

Code	Open		Volume		Amount	
	Mean	Std	Mean [× 10^4^]	Std [× 10^4^]	Mean [× 10^5^]	Std [× 10^5^]
600276.SH	73.34	16.59	22.06795	15.51354	16.57419	11.52752
600763.SH	117.45	102.49	3.606954	2.158886	4.267753	4.771402
300122.SZ	73.3	57.06	12.57051	7.281096	11.05719	12.44737
600085.SH	30.37	3.7	10.00756	9.440757	3.143793	3.304401
000423.SZ	45.64	12.32	6.319308	3.850551	2.928001	2.047151
601607.SH	20.97	2.71	14.77902	12.0502	3.115376	2.643739
300003.SZ	28.44	6.47	18.73861	11.65405	5.566067	3.819116
002038.SZ	21.31	10.08	8.79172	7.822409	1.760333	1.613011
300244.SZ	26.96	7.39	7.283911	5.086287	2.110358	1.930123
000963.SZ	40.75	17.95	19.10332	20.31588	6.698094	8.077395
000931.SZ	7.79	1.31	11.28132	12.79515	0.958011	1.216137
600196.SH	37.95	13.34	32.12141	28.10605	14.31098	17.73849
603883.SH	63.14	13.81	1.972923	1.328842	1.32386	1.098693
930767.CSI	5188.66	773.37	11075.73	3960.734	1621103	831315.5

**Table 2:** Model description.

Abbreviation	Description
AGKN	Attention-based graph kernel network
AGKN-K	AGKN without the kernel method (K)
AGKN-G	AGKN without the graphing learning method(G)
AGKN-S	AGKN without stock-axis attention(S)
AGKN-T	AGKN without time-axis attention(T)
A-BiLSTM	An attention-LSTM model (state-of-the-art model) [34]
LSTNet	A deep neural network, which combines convolutional and recurrent neural network [35]
KNN	A model which integrates the kernel method and deep neural networks [31]

**Table 3:** Experiment results.

Model	MSE	RMSPE
AGKN	17.4042	0.0480
AGKN-K	19.1892	0.0492
AGKN-G	46.8264	0.0786
AGKN-S	19.7096	0.0580
AGKN-T	17.7034	0.0482
A-BiLSTM	44.4000	0.0915
LSTNet	34.3967	0.0915
KNN	1889.5324	0.5664

## Data Availability

The data used to support the findings of this study are available at https://tushare.pro/.
